# A case of the world’s largest renal cell carcinoma

**DOI:** 10.1002/iju5.12236

**Published:** 2020-10-25

**Authors:** Kotaro Takeda, Gina Murray, Nasreen Vohra, John T Fallon

**Affiliations:** ^1^ Department of Pathology and Laboratory Medicine Brody School of Medicine East Carolina University Greenville North Carolina USA; ^2^ Division of Surgical Oncology Department of Surgery Brody School of Medicine East Carolina University Greenville North Carolina USA

**Keywords:** gigantic renal cancer, kidney cancer, nephrectomy, papillary renal cell carcinoma type 1, renal cell carcinoma

## Abstract

**Introduction:**

Renal cell carcinoma is often discovered at an early stage due to the increased use of imaging studies in the current era; therefore, its presentation as a gigantic renal cell carcinoma is rarely encountered.

**Case presentation:**

A 59‐year‐old male presented to our hospital due to dizziness, fatigue, and increasing abdominal distension. A computed tomography scan showed an extremely large mass occupying most of the abdomen and pelvis. Surgical resection of the mass was performed. The largely cavitary mass with fibrous capsule was 43 cm and 13.0 kg, and contained a large amount of necrotic tissue. A portion of the left kidney was identified at the periphery of the mass, indicating that the tumor was arising from the left kidney. The final pathologic diagnosis was type 1 papillary renal cell carcinoma.

**Conclusion:**

To the best of our knowledge, this tumor is the world’s largest malignant renal tumor.

**Abbreviations & Acronyms**
CT: computed tomography
NA: not available
RCC: renal cell carcinoma
SFT: solitary fibrous tumor

**Keynote message**

RCC is often discovered at an early stage and presentation as a huge mass is rarely encountered nowadays. We present a case of a gigantic type 1 papillary RCC.

## Introduction

RCC is often discovered at an early stage due to the increased use of imaging studies in the current era; therefore, presentation as a huge mass is rarely encountered.^1^ However, if renal tumors are left untreated, they can grow to huge sizes.^2^, ^3^ We report a case of an extremely large RCC. To the best of our knowledge, this tumor is the world’s largest malignant renal tumor.

## Case presentation

A 59‐year‐old male presented to our hospital due to dizziness, fatigue, and increasing abdominal distension. He was 123 kg. A physical examination found markedly distended abdomen and temporal and extremity muscle wasting, along with tachycardia and tachypnea (blood pressure 100/66 mmHg, heart rate 112/min, and respiratory rate 24/min). Laboratory tests showed anemia (hemoglobin 8.1 g/dL), hypoalbuminemia (albumin 2.6 g/dL), and renal failure (creatinine 3.44 mg/dL). Tumor makers (CA19‐9 and carcinoembryonic antigen) were within normal range.

**Fig. 1:**
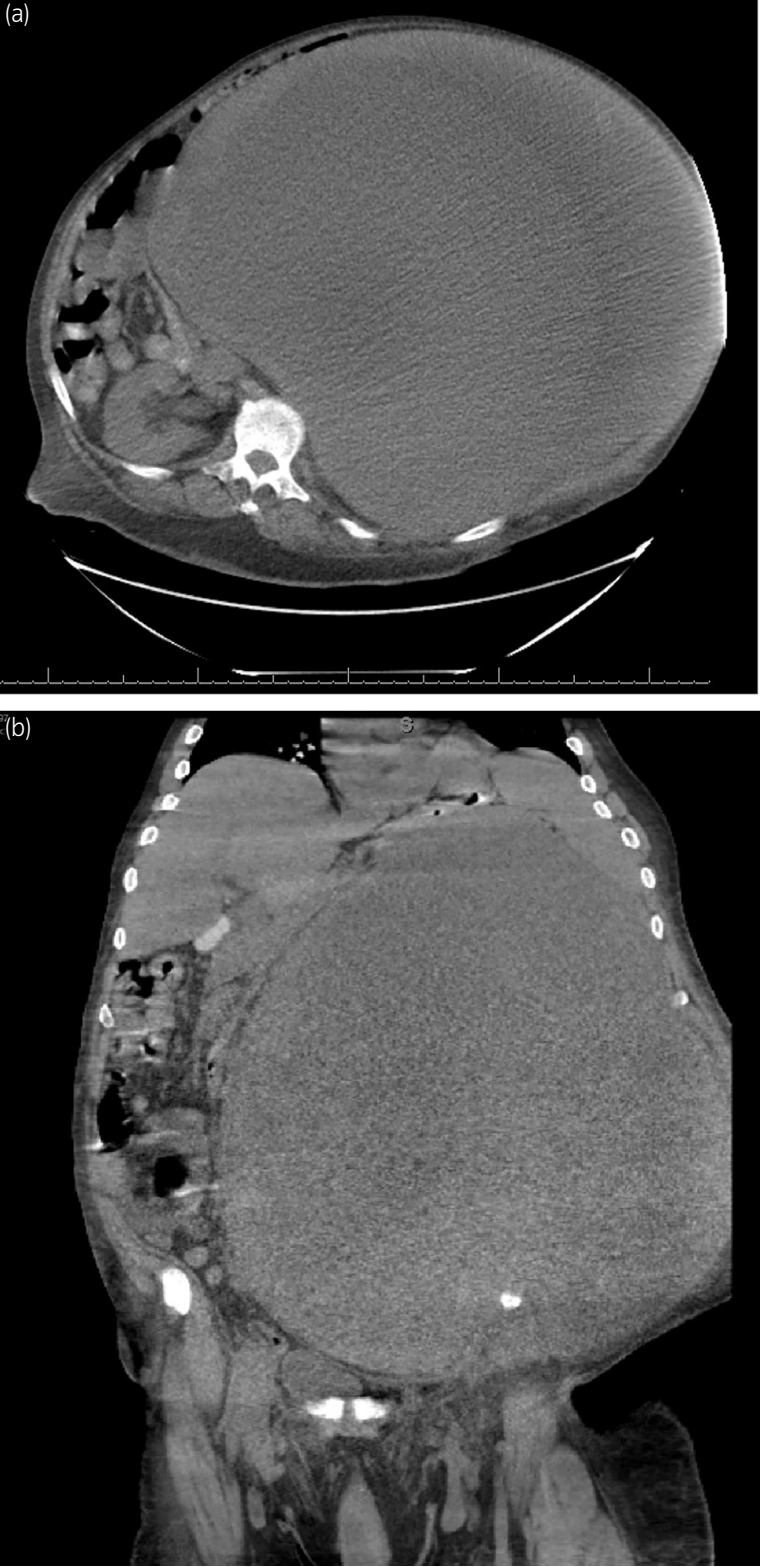
Abdominal CT images (a: axial view, b: coronal view) revealed a huge mass occupying most of the abdomen and pelvis.

Abdominal CT scan revealed a 43 cm extremely large mass occupying most of the abdomen and pelvis (Fig. 1a,b). The left kidney was not identified. The mass contained mixed densities of a combination of fluid and soft tissue component. No masses were seen in chest CT.

Due to the large size of the mass, he resulted in limited oral intake and was started on enteral feeds. However, he failed to thrive, and became hypotensive with melena. An esophagogastroduodenoscopy showed some blood in the stomach; however, a celiac angiogram showed no evidence of active bleeding. He was intubated and started on pressors and blood transfusion. The decision was made to explore the patient urgently. Upon entry, the transverse to left colon (splenic flexure) was ischemic, and this portion was resected. A large retroperitoneal mass was noted. The mass was cystic with murky foul‐smelling fluid in it. During dissection of the mass, there was an area of weakness that spontaneously ruptured with extrusion of the cyst contents of the mass all over the abdominal cavity and the retroperitoneum as well as spillage onto the floor. A significant amount of the fluid in the cyst was suctioned out. Once the mass was partially decompressed, the opening in the mass was repaired to allow control of the spillage. Ultimately the mass was resected after dissecting it free from is attachments. On further exploration, a perforated anterior duodenal ulcer was noted, which was repaired.

**Fig. 2:**
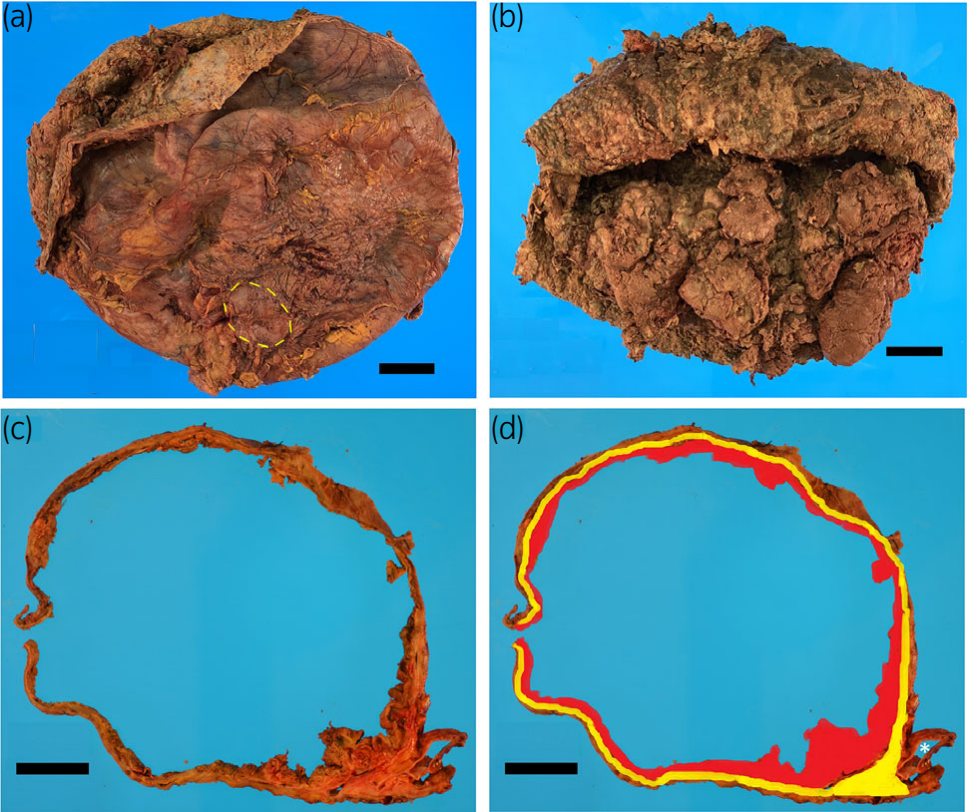
Macroscopic pictures of resected mass (a: outside view, b: inside view). The mass with fibrous capsule was largely cavitated and contained a large amount of necrotic tissue. A portion of the left kidney was identified at the periphery of the mass (a, yellow dot line). Cross section of the mass and the left kidney (c) and its diagram (d). The red and yellow areas are where carcinoma and benign renal parenchyma were present, respectively (d). Note that the carcinoma is completely surrounded by benign renal parenchyma and no invasion to the renal sinus (right lower corner; asterisk, left ureter). Scale bar, a–d: 5 cm.

**Fig. 3:**
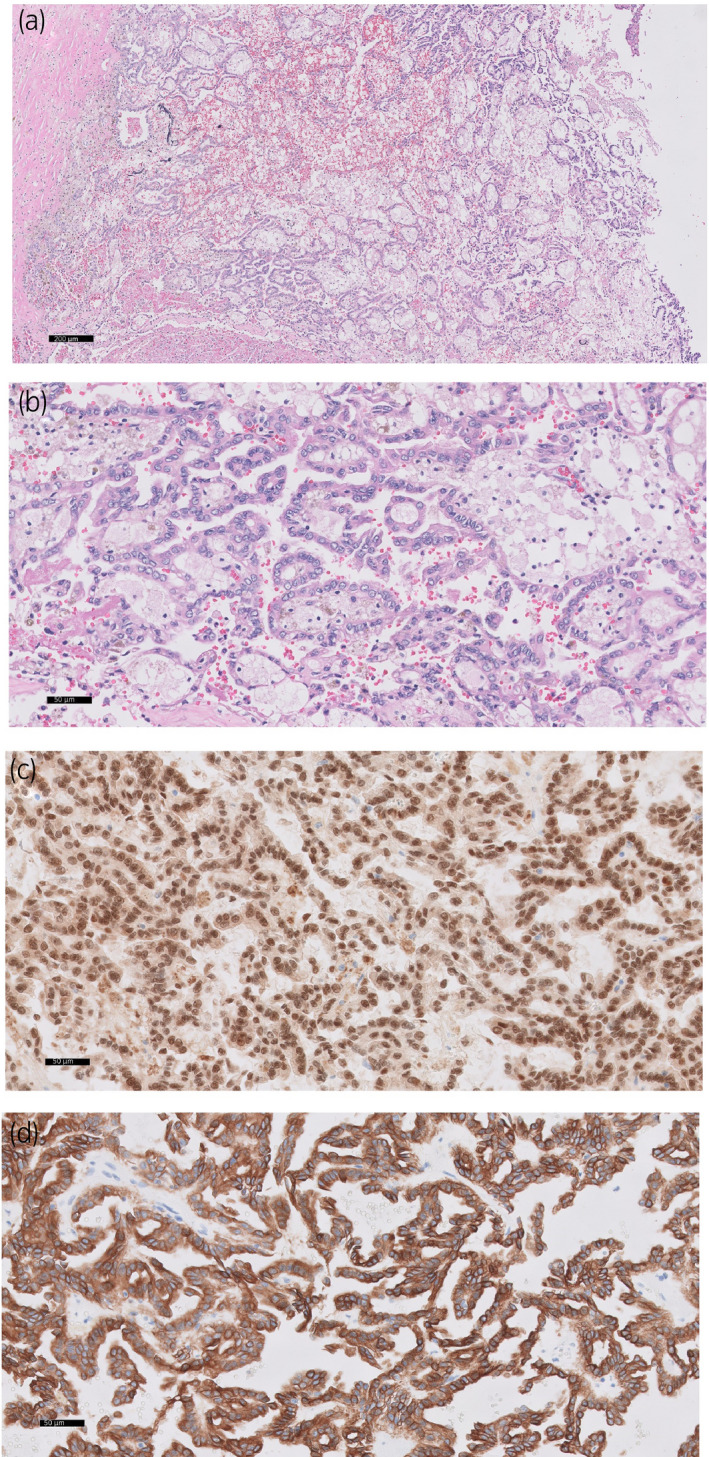
Photomicrographs of the mass (a: low magnification, b: high magnification) showed papillary structures lined by single layer of low‐grade tumor cells and contained foamy macrophages (hematoxylin and eosin stain, scale bar, a: 200 µm, b: 50 µm). Immunohistochemical studies of PAX8 (c) and cytokeratin 7 (d) revealed strong positivity in tumor cells (scale bar, c and d: 50 µm).

The largely cavitary mass with fibrous capsule was 43 × 42 cm and 13.0 kg (a sum of suctioned content and spillage during the surgery and the resected specimen) and contained a large amount of necrotic tissue (Fig. 2a,b). A portion of the left kidney was identified at the periphery of the mass (Fig. 2a, yellow dot line). Despite the extremely large size, the tumor was confined to the kidney and no invasion into the perirenal adipose tissue or renal sinus was identified (Fig. 2c,d). Microscopically, the tumor consisted of papillary structures lined by single layer of tumor cells with low‐grade nuclear features and contained foamy macrophages (Fig. 3a,b). Nucleoli were appreciated at the higher magnification. Importantly, a rim of atrophic renal parenchyma was circumferentially appreciated, confirming renal origin. A panel of immunohistochemistry revealed tumor cells; positive for PAX8 (Fig. 3c), cytokeratin 7 (Fig. 3d), vimentin, and CD10. The final pathologic diagnosis is type 1 papillary RCC with nuclear grade 2.

Postoperative cure was complicated requiring tracheostomy and mechanical ventilation. He developed intra‐abdominal abscess requiring drainage and antibiotics in the intensive care unit. Due to his ongoing deterioration and minimal hope of recovery, comfort care measures were initiated. He deceased 1 month later.

## Discussion

We performed extensive literature search using the PubMed database for case reports of a large renal tumor published in the English language. Table 1 summarizes cases of extremely large malignant renal tumor including our case.^4^, ^5^, ^6^, ^7^, ^8^, ^9^, ^10^, ^11^, ^12^ Overall, large renal tumors are malignant neoplasms, such as RCC subtypes, malignant SFT, and leiomyosarcoma, with benign angiomyolipoma being an exception.^5^ Currently, the largest reported renal tumor is 11.5 kg, a chromophobe RCC reported in 2009.^4^ To the best of our knowledge, our 13.0 kg RCC is the world’s largest renal tumor.

**Table 1:** Summary of case reports of large malignant renal tumor published in the English language

Case	Year	Age	Sex	Size (cm)	Weight (kg)	Diagnosis	Outcome (Follow up)	Reference
1	2007	42	M	28	NA	Sarcomatoid RCC	Unknown	6
2	2009	55	M	35	11.5	Chromophobe RCC	Alive (20 months)	4
3	2010	54	M	25	2.5	Clear cell RCC	Alive (1 year)	8
4	2012	72	M	20	1.85	Malignant SFT	Alive (9 months)	12
5	2013	48	M	22	2.7	Clear cell RCC	Deceased (6 months)	11
6	2014	66	F	23	NA	Malignant SFT	Alive (9 months)	10
7	2016	75	M	28	NA	Papillary RCC	Unknown	7
8	2016	52	M	23	3.63	Leiomyosarcoma	Unknown	9
9	2020	59	M	43	13.0	Papillary RCC	Deceased (1 month)	This case

The histologic type of this tumor is papillary RCC type 1. Papillary RCC has been subdivided into type 1 and type 2.^13^ Type 1 RCC has papillae covered by a single layer of cells with lower nuclear grade, in contrast, type 2 RCC is characterized by papillae with pseudostratification of cells with higher nuclear grade and oncocytic cytoplasm.^13^ In general, papillary RCC is associated with a favorable outcome; moreover, type 1 papillary RCC has an even better prognosis than type 2 papillary RCC.^13^ It is assumed that being a low‐grade carcinoma likely allowed clinically indolent and expansile growth up to such a large size while remaining confined within the kidney. Therefore, even though it was gigantic in size, the tumor had a low pathologic stage, pT2b (American Joint Committee on Cancer 8th edition).^14^

It is not clear why there is no distant metastasis in spite of the large size; however, several possibilities are considered. First, there is no definitive tumor extension into major renal vein or its segmental branches macroscopically as well as lymphovascular invasion microscopically despite extensive search. Second, there is no invasion to the renal sinus or perirenal adipose tissue where blood vessels are abundant. The tumor has been surrounded by fibrous capsule and well circumscribed. Third, the tumor shows expansile growth but not infiltrative growth. Lastly, the tumor is low‐grade carcinoma. Generally, low‐grade carcinoma less likely metastasize compared to high‐grade one.

Surgery is curative in the majority of patients with RCC without metastasis.^15^ Patients with large RCC are associated with poorer prognosis, because nearly 40% of the patients have metastasis at diagnosis.^8^ Surgery is therefore the preferred treatment for patients with gigantic RCC if metastasis is excluded. Preservation of capsule of gigantic renal mass ensures complete resection.^7^ The cause of death in our case is due to the ultimate nature of the tumor (mostly necrotic and liquefied cystic mass with an area of weak wall) as well as complicated presurgical conditions (malnutrition, anemia, ischemic bowel, duodenal ulcer perforation, and hypotension).

## Conflict of interest

The authors declare no conflict of interest.
